# Pre exposure to enriched environment alleviates brain injury after ischemia-reperfusion by inhibiting p38MAPK/STAT1 pathway

**DOI:** 10.1007/s11033-022-08184-5

**Published:** 2022-12-27

**Authors:** Xin-Ya Shen, Yu Han, Zhen-Kun Gao, Ping-Ping Han, Xia Bi

**Affiliations:** 1 Shanghai University of Traditional Chinese Medicine, 201203 Shanghai, China; 2 Shanghai University of Sport, 200438 Shanghai, China; 3 Shanghai University of Medicine & Health Sciences Affiliated Zhoupu Hospital, 201318 Shanghai, China

**Keywords:** Enriched environment, MAPK, p38MAPK/STAT1, Inflammation, Ischemic stroke, MCAO

## Abstract

**Background:**

Stroke is one of the major diseases that endangers human health. It is widely reported that enriched environment (EE) can improve the neurological function in different brain injury models. Recently, relevant researches have indicated that MAPK pathway is closely related to the inflammatory response in nervous system related diseases. However, whether pre exposure to EE (EE pretreatment) has a preventive effect, and its mechanism are not clear. Therefore, this study aimed to determine the possible benefits and related mechanisms of EE in preventing brain injury after acute ischemia-reperfusion.

**Methods:**

Adult Sprague Dawley rats were kept in enriched or standardized environments for 21 days. Then the middle cerebral artery of rats was occluded for one hour and 30 min, and then reperfusion was performed. Then their neurological deficit score was evaluated. Cerebral edema, along with ELISA and protein quantities of p38MAPK, JNK, ERK, IL-1β, TNF-α, and co-localization of Iba1 were assessed. Changes in neuroinflammation and apoptosis were also detected in the penumbra cortex.

**Results:**

Our research showed that EE pretreatment significantly alleviated acute cerebral ischemia-reperfusion injury in rats. Including the reduction of brain edema and apoptosis, and the improvement of neurological scores. In addition, the protein level of p38MAPK was significantly down regulated in EE pretreatment group, and the downstream protein STAT1 had the same trend. In addition, immunofluorescence results showed that Iba1 in EE pretreatment group decreased, the ELISA results showed that the classical proinflammatory cytokines increased significantly, while anti-inflammatory cytokines in EE pretreatment group increased, and the same results were obtained by Western blot analysis.

**Conclusion:**

On the whole, our research demonstrated that EE pretreatment can have a protective effect on the organism by inhibiting the p38 MAPK/STAT1 pathway. Thus, EE can be one of the most promising means of disease prevention. Secondly, p38MAPK/STAT1 pathway may be a latent target for the prevention of acute ischemic stroke.

## Introduction

Stroke, as a cerebrovascular disease represented by ischemia or hemorrhage, has a high incidence rate. Most patients will have different degrees of disability, and even die. [1, 2]. The recombinant tissue plasminogen activator (Rt-PA) is a commonly used clinical treatment, but the narrow time window limits its large-scale application, and many people have not received timely and effective treatment [3]. Therefore, it is urgent to develop new prevention or treatment methods for brain injury caused by ischemic stroke.

**Fig. 1:**
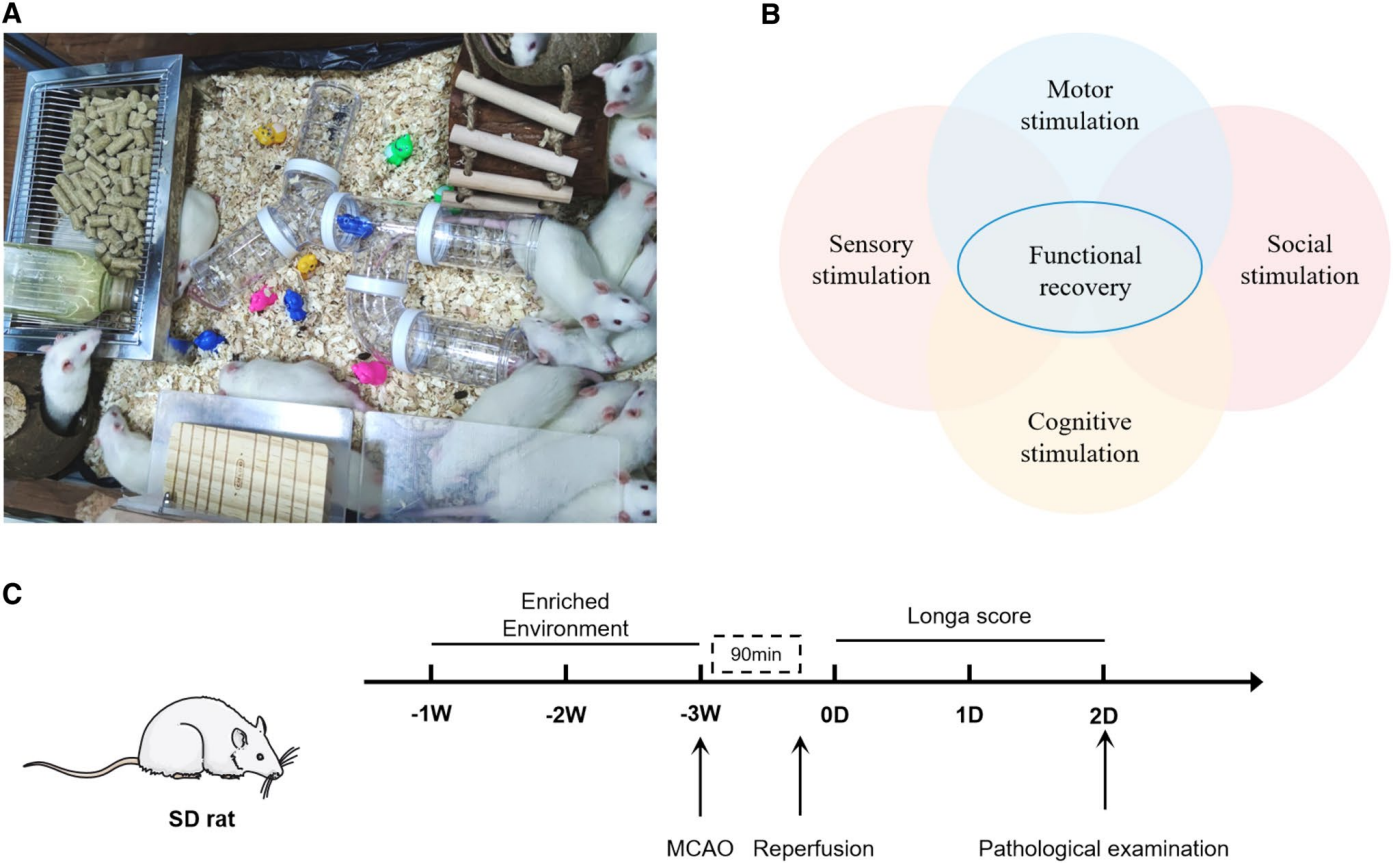
Enriched Environment (EE) scene layout and experiment schedule. **A** The scene settings of the EE. Arch bridges, pipes and tree holes for exploration and movement; Decorations and toys of different colors provide visual stimulation; The rich environment has larger space and more members to provide social stimulation. In addition, items are replaced once every three days with their placement to retain the freshness of the environment. **B** Stimulation mode of EE. **C** Timeline of the experimental procedure

The massive research indicated that giving multisensory stimuli (such as motor, visual, auditory, etc.) after cerebral ischemia injury can be effective in ameliorating the neurological dysfunction of patients [4]. The enriched environment (EE) is a noninvasive intervention modality that includes multifaceted stimuli such as motor stimulation, cognitive stimulation, social stimulation, and sensory stimulation [5] (Fig. 1A, B). Compared with the standard environment (SE), the EE has small toys with different functions and shapes, and a larger activity space for exploration. Secondly, more participating members in the space provide social stimulation, and items will be changed regularly to maintain the novelty of the environment. In the EE, we can have more opportunities to receive multiple stimuli and provide a favorable environment for the repair of the body. In related studies on neurological impairment, the recovery of brain injury after EE intervention has confirmed this view [6, 7]. Accumulating evidence suggests that exposure to EE after brain damage can ameliorate neurological outcome by reducing cell death, promoting motor function recovery, promoting neurogenesis, reducing microglial activation, and altering gene expression. However, there are still few relevant studies on whether EE pretreatment intervention can play a protective role and what its specific mechanism is.

After ischemic stroke, the brain tissue lacks necessary oxygen and energy, resulting in the disorder of neuronal metabolism, the destruction of ion balance and cell homeostasis, and eventually neuronal apoptosis occurs and the formation of infarct core and ischemic penumbra [8]. A series of cascades such as inflammation and apoptosis are triggered. It further damages neurons, endothelial cells, and glial cells, exacerbating ischemic penumbra injury [9, 10]. Inflammation is essential throughout the pathological process of ischemic stroke. After brain injury, the inflammatory response profoundly influences disease progression and recovery. The activation of glial cells, mainly microglia, is the first manifestation of the inflammatory response. Neurotoxic substances such as inflammatory cytokines, glutamate and nitric oxide can be induced by activated microglia [11, 12]. In addition to this, microglia can induce the release of proinflammatory cytokines [13]. Numerous studies have shown that cerebral ischemia induces a severe neuroinflammatory response, manifested by an increased number of inflammasomes and proinflammatory cytokines [14]. Importantly, inhibiting inflammation can significantly attenuates brain damage following ischemia-reperfusion [15, 16]. However, the specific pathway through which different intervention methods inhibit inflammation has not yet been determined. There are still a lot of studies devoted to this, and new mechanisms are constantly added.

The mitogen activated protein kinases (MAPKs), as a group of serine-threonine protein kinases that can be activated by various stimuli, are mainly divided into three subfamilies: p38, JNK and ERK, which mediate fundamental biological processes and cellular responses to external stimuli [17, 18]. P38 and JNK are both related to inflammation and apoptosis. p38 is mainly used in the research of inflammation, while JNK is mainly focused on apoptosis; ERK is related to cell growth and differentiation, and mainly affects the proliferation, migration, and invasion of cancer cells. MAPK has been widely studied in inflammation [19]. Among them, p38MAPK signaling is closely related to ischemic injury mediated by inflammation, and inhibition of p38MAPK signaling can inhibit neuronal apoptosis and inflammation response, protect damaged brain tissue, and ameliorate neurological injury [20]. The main inducement of p38MAPK is stress response such as hypoxia and ultraviolet radiation, and it is participate in governing the synthesis of inflamentary transmitter at the transcriptional as well as translational levels [21]. It is a potential target for anti-inflammatory treatment. So far, the activation mechanism of p38MAPK pathway has been well studied. The p38MAPK cascade, as a central signal pathway, is mainly responsible for transmitting stimuli and other signals to various targets in the cytoplasm and nucleus. Some secondary signal molecules, such as STAT1, STAT3 and STAT4, are located downstream of p38MAPK [22]. Especially, p38mapk can induce activation of STAT1. An increasing body of evidence suggests that that p38MAPK/STAT1 pathway plays a significant role in the inflammatory response[23]. p38MAPK signaling pathway is also participated in the pathological process of stroke, which has been validated in numerous cellular and animal experiments [24–27]. However, it is not clear whether the p38MAPK/STAT1 pathway is associated with the protective effect of EE pretreatment. In addition, the effect of EE pretreatment on p38MAPK/STAT1 pathway still unclear.

In our study, we proposed that EE could also attenuate brain injury after acute ischemic stroke via p38MAPK/STAT1 pathway. More importantly, we placed the intervention time of EE before modeling to investigate whether advance EE exposure had a protective effect on the brain. We ascertained the influences of EE pretreatment on p38MAPK/STAT1 signaling pathway and inflammatory related cytokines in the contralateral penumbra cortex of adult rats subjected to middle cerebral artery occlusion /reperfusion (MCAO/R) by Western blotting. Our results show that EE pretreatment can inhibit inflammatory response through p38MAPK/STAT1 pathway, thereby reducing brain damage caused by acute ischemic stroke. More importantly, this research result has certain clinical significance, that is, pleasant mood and relaxed atmosphere are conducive to the prevention and cure of diseases.

## Materials and methods

### Experimental animals

The animals used in the experiment were adult male Sprague Dawley rats(weighed 230–270 g and aged 6–8 weeks old), which were purchased from Shanghai SLRC Laboratory Animal Co., Ltd. The rats were raised under standard experimental conditions and experienced 12 h light and 12 h dark cycle. Food and water can be freely obtained, and animals can move freely. The experimental process conforms to the guidelines for the care and use of experimental animals of the National Institutes of Health (Promulgated by the National Research Council in 1996) and has also been approved by the Nursing Committee of the Experimental Animal Institution of Shanghai University of Traditional Chinese Medicine to minimize the pain of animals (Shanghai, China; permit number: 20,170,005,047,368).

### Surgical procedure

After 3 weeks of intervention, rats underwent MCAO or sham operation. As in the previous experimental procedure, the right middle cerebral artery occlusion (tMCAO) was briefly blocked to establish an animal model of ischemic stroke [28]. In short, rats were first anesthetized with 1.5% isoflurane and then were placed on the circulating heating pads to maintain the temperature of animals within the proper range. The rats were placed on the operating platform face up. After preserved skin at operation site, an incision of about 2 cm is made at the surgical site. Find the common carotid artery (CCA), internal carotid artery (ICA) and external carotid artery (ECA) on the right side of the rat after blunt dissection. Push the appropriate size nylon monofilament (Rayward Life Technology Co., Ltd., Shenzhen, China) from ECA to ICA chamber. After the middle cerebral artery was blocked for 90 min, nylon monofilament was taken out to restore blood flow. The rats in Sham group did not really insert nylon monofilament, and other procedures were the same as those in MCAO group. During operation, we used laser speckle Contrast imaging(LSCI) (Moor Instruments, Devon, USA) to detect the cerebral blood flow (CBF) on the ischemic side of rats. Rats were scored according to Longa scale [29] after they were resuscitated from anesthesia. Only the rats with a score of 1–3 can be considered as successful models and used for further experiments.

### Experimental groups and EE paradigm

According to the purpose of the experiment, the animals were randomized into the following groups: EE group, SE group and sham group (14 rats in each group). We redesigned the experimental cage according to the literature and international requirements (Fig. 1A). There are various small objects in it for various stimuli, including hanging (rope) ladder, transparent partition, running wheels, seesaw, tunnels, coconut nests and models of small animals of different colors for rats to explore; There are also audible and luminous spheres for multi sensory stimulation; Up to 14 rats can be housed in each cage. The placement of items within the cage was transformed every 3 days to keep the freshness of the environment. The standard environment is a common cage size, which can only accommodate 4 rats for free movement, and there are no other items except basic water and food.

### Behavioral testing

Assessment of neurological deficits in MCAO rats with the Longa scale. After tMCAO operation, all rats were tested on day 0, 1 and 2 of reperfusion, and the operator was known nothing about the experimental test group. The Longa scale was applied to evaluate neurological deficit in rats: 0, no neurological symptoms; 1, inability to fully extend the front paw on the paralyzed side; 2, turning around to the paralyzed side when walking; 3, tipping to the paralyzed side when walking; 4, unconscious, unable to walk **(**Table 1**)**.

### Brain Water Content Measurement

Two days after the operation, the post-ischemic brain edema was estimated according to the following formula: (wet weight - dry weight)/wet weight × 100% [30]. In general, brain tissue was collected immediately without cardiac perfusion, then wet weight was measured separately using an analytical balance, and finally dry weight was measured after drying at 105 °C for one day.

### Western blotting assay

Collected infarcted cortex tissue of rats, sonicate it in the homogenization buffer. We evaluated the concentration of protein by dinyltetraketo acid method. First, mix the sample with its buffer, heat it in boiling water at 95 º C for 10 min, separate the protein sample by polyacrylamide gel electrophoresis, and then transfer the protein on the gel to PVDF membrane. Finally, the membrane was incubated with the secondary antibody overnight at 4 °C. p-p38MAPK (1:100 dilution, Santa, sc-166,182), p38MAPK (1:1000 dilution, Cell signaling, 8690), STAT1 (1:5000 dilution, abcam, ab109461), TNF-α (1:900 dilution, abcam, ab66579), IL-1β (1:1000 dilution, Bioworlde, BS6067). Wash three times with buffer, then label the secondary antibody with horseradish peroxide at room temperature. Image observation using EPSON imaging system (EPSON; V300, Japan). Densitometric analysis was performed using Alpha Software (Alpha Innotech; alphaEaseFC, America).

### TUNEL assay

To detect neuronal apoptosis around penumbra after ischemic injury, we used TdT mediated dUTP nick end labeling (TUNEL) staining. In short, the tissues were immediately fixed with the newly prepared 4% formaldehyde at room temperature overnight. After dehydration, paraffin embedded tissues were made into slices, and paraffin was removed after heating. After repeated rinsing, treat it with 0.5% H2O2 solution at room temperature for 20 min. Incubate the slices with TdT reaction buffer solution and rinse again. The labeled cells were treated with DAB solution and stained with hematoxylin. Finally, fluorescence microscopy (Vectra 3, PerkinElmer, United States) was used to observe the sections (Vectra 3, PerkinElmer, USA) and calculate the number of TUNEL positive cells. Randomly select at least six visual fields and calculate the average percentage to represent the final data. Percentage of apoptotic cells (%)=(TUNEL + cells/total cells)×100%. Apoptotic cell death was determined by counting the number of positively stained cells using the TUNEL index.

### HE staining

Using HE staining to observe the pathological changes of cortical tissue after ischemia. Deparaffinization and hydration were performed first, stained with hematoxylin for 5 min and then rinsed off with tap water. The samples were differentiated with 0.1% hydrochloric acid/ethanol, and excess dye was rinsed with clean water. The samples were then immersed in eosin dye for 2 min. After washing, it was dehydrated with absolute alcohol and sealed. The histopathological characteristics of the samples in each group were observed using optical microscope (Nikon, Tokyo, Japan).

### Enzyme-linked immunosorbent assay (ELISA)

The inflammatory mediators in the ischemic penumbra were detected by ELISA kit. After the rats were killed, the tissues were collected, and the target sites were removed by microanatomy under low temperature environment. After homogenization with physiological saline, centrifugation was carried out, and the supernatant was extracted for standby [31]. According to the instructions provided by the manufacturer, add the supernatant into the kit to test the relevant anti-inflammatory and pro-inflammatory factors.

### Immunofluorescence staining

After the rats were killed, they were perfused and fixed in 4% paraformaldehyde for two days. The tissue was then cut into 50 µ m coronal sections. The tissue slides were cleaned with TBS solution for 10 min and sealed with sealing solution (10% serum + 1% BSA) + 0.3% triton-100* at room temperature for 2 h. After cleaning again for 15 min, the tissue slides were incubated with Iba-1 antibodies at 4 ℃ overnight. After washing, place it on the glass slide to dry, and finally use 90% glycerin seal. Fluorescent microscope was used to observe and take images, and ImageJ was used for cell counting.

### Statistical analysis

The data obtained in this experiment are normally distributed and uniformly expressed as mean ± standard error of the mean (SEM). Single factor analysis of variance and Tukey multiple comparison were used to determine the comparison between groups. A p value of at least＜0.05 was considered statistically significant. IBM SPSS statistics 26 software was used in the study.

## Results

### Successfully established cerebral ischemia reperfusion model

**Fig. 2:**
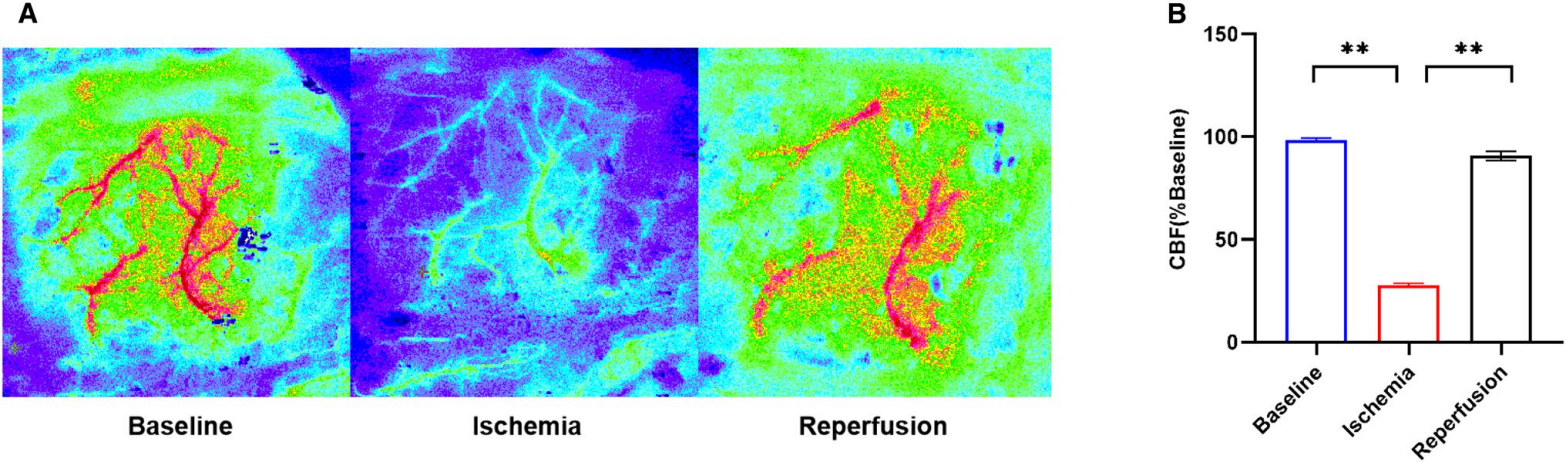
Successfully established cerebral ischemia reperfusion model. The image and quantization results obtained by LSCI measurement (**A** and **B**). Data were presented as mean ± SEM. **p < 0.01

To verify the correct establishment of ischemic stroke model, we ground and exposed the skull bones of experimental rats to the laser and examined their cerebral blood flow (CBF) using LSCI at three time points: before wire plug insertion, after wire plug insertion, and after reperfusion. Analyzing the results of LSCI revealed that when the rat had a wire plug inserted and the model was successfully established, its CBF decreased to less than half, whereas it recovered to nearly 86% after reperfusion (p < 0.01). (Fig. 1A, B). Therefore, the rat MCAO model in this experiment successfully simulated the process of ischemia-reperfusion (Fig. 2.

### EE pretreatment protects brain against acute ischemia reperfusion induced injury

**Fig. 3:**
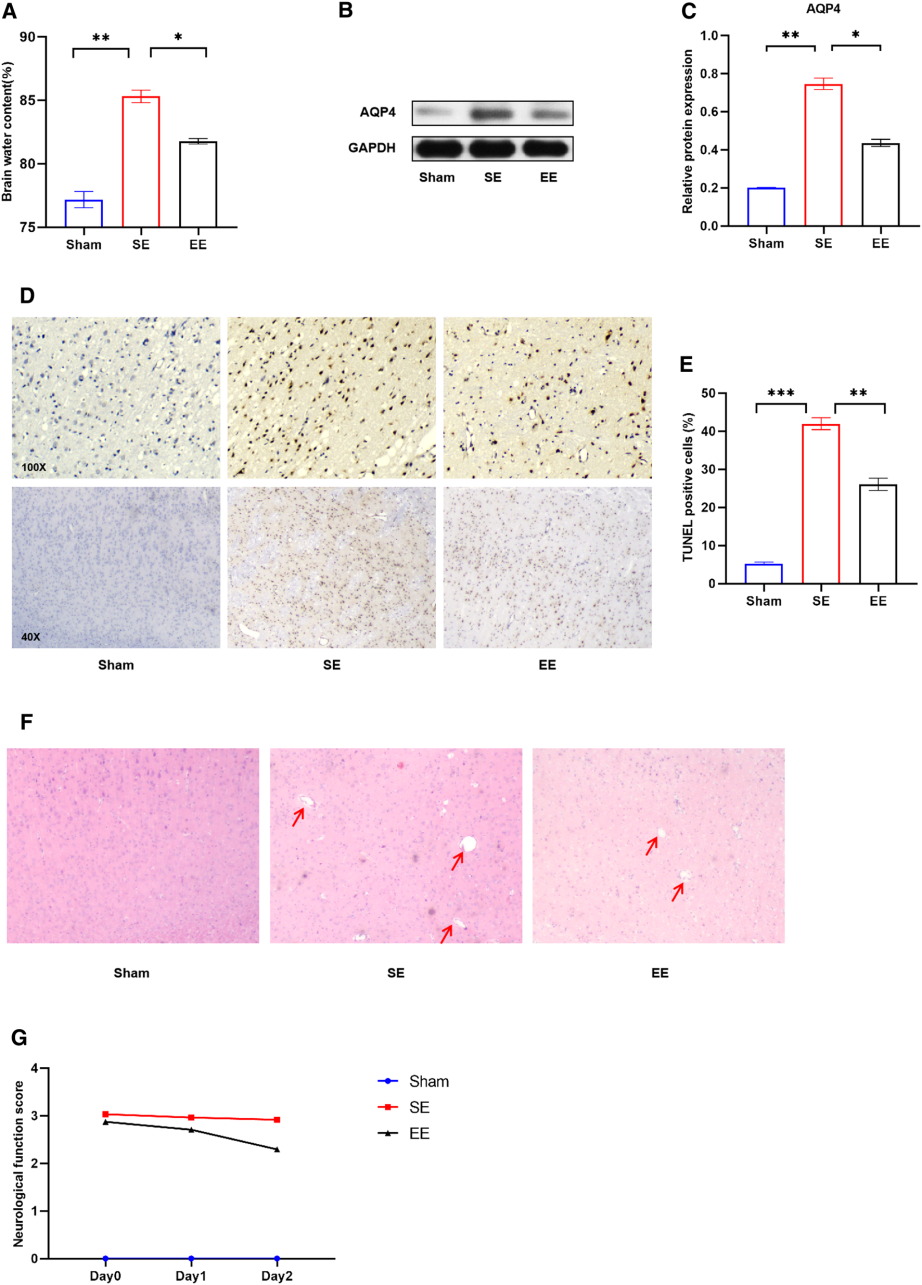
EE pretreatment alleviated brain injury after ischemia-reperfusion in rats. **A** Brain water content measurement. **B**, **C** Representative western blot (**B**) and quantitative results of the levels of AQP4 (**C**) in the cortex of different experimental groups; n = 6. Data are expressed as mean ± SEM. One-way analysis of ANOVA with Bonferroni’s post hoc test was used. **p < 0.01,***p < 0.001. **D** Representative images of TUNEL assay of cortical tissue in different groups were enlarged 100 and 40 times to show the apoptosis levels (Black: apoptotic cells. Blue:nucleus). **E** Quantitative anlysis of the apoptosis index percentage. Apoptosis index was caculated at the ratio of (TUNEL-positive cells)/(total cells) ×100% n = 6; mean ± S.D.; **P < 0.01. **F** HE staining results showed that the cells in the sham group were arranged regularly and the structure was clear. Se group was obviously damaged, with fuzzy structure and irregular arrangement. Compared with Se group, the pathological changes in EE group were significantly improved. **G** Longa scores were assessed at day 0, day1 and day 2 after. n = 6. Data were presented as the mean ± SEM. (Color figure online)

In order to visually observe the brain injury between different groups, we measured the brain edema of rats. After the rats were sacrificed, the brains were rapidly separated into ischemic and non ischemic sides. The brain sections on both sides were packed with tin foil, and the wet weight was measured directly with a balance, whereas the dry weight needed to be measured after drying at 105 °C for 24 h. The post-ischemic brain edema was estimated according to the following formula: (wet weight - dry weight)/wet weight × 100%. At 48 h post-reperfusion, EE pretreatment significantly decreased brain water content compared with the SE group (Fig. 3A). To further confirm this result, we detected aquaporin 4 (AQP4) using Western blot analysis. AQP4 is a bidirectional water transporting protein mainly responsible for water transport and regulation of brain water homeostasis [32]. In previous ischemia-reperfusion experiments, AQP4 null mice had less brain edema and infarct volume than control mice [33]. Consistent with the previous results, the level of AQP4 in SE group rats increased significantly after MCAO. However, compared to the SE group, the protein levels in the EE group were significantly decreased (Fig. 3B, C). This indicates that rats pretreated with EE have stronger resistance, and the lower AQP4 level confirms this conclusion.

In addition, to further determine whether EE can mitigate brain damage, we used TUNEL method to detect apoptotic cells in ischemic penumbra. After successful modeling, a large number of TUNEL positive cells appeared in brain slices of SE rats, while EE pretreatment group significantly inhibited this phenomenon (Fig. 3D, E). Finally, we also observed the pathological characteristics of tissues among groups using HE staining (Fig. 3F). The results illustrated that the cells in the sham group were arranged regularly and had a clear structure. In SE group, the damage was obvious, the structure was blurred, and the arrangement was irregular. Compared with SE group, the pathological changes in EE group were significantly improved. Thus, pretreatment with EE significantly attenuated brain injury after ischemia-reperfusion. Similarly, similar results were obtained with the neurological deficit score. The overall functional performance of rats in EE group was better than that in control group (Fig. 3G).

Overall, EE pretreatment exhibited significant neuroprotective effects in ischemic stroke, but the underlying mechanisms need to be further investigated.

### EE inhibited the expression of inflammatory related pathways after acute ischemia reperfusion

Next, we sought to investigate the molecular mechanisms by which EE pretreatment attenuates brain damage after stroke. The abnormality of MAPK signal pathway is related to many human diseases. Sustained activation of the p38 signaling pathway has been shown to be critically involved in the progression of Alzheimer’s disease [34]; However, there are few studies on MAPK in ischemic stroke at present [35]. Whether the protective effect of EE pretreatment is related to it and the downstream pathway mediating this effect are still unclear.

**Fig. 4:**
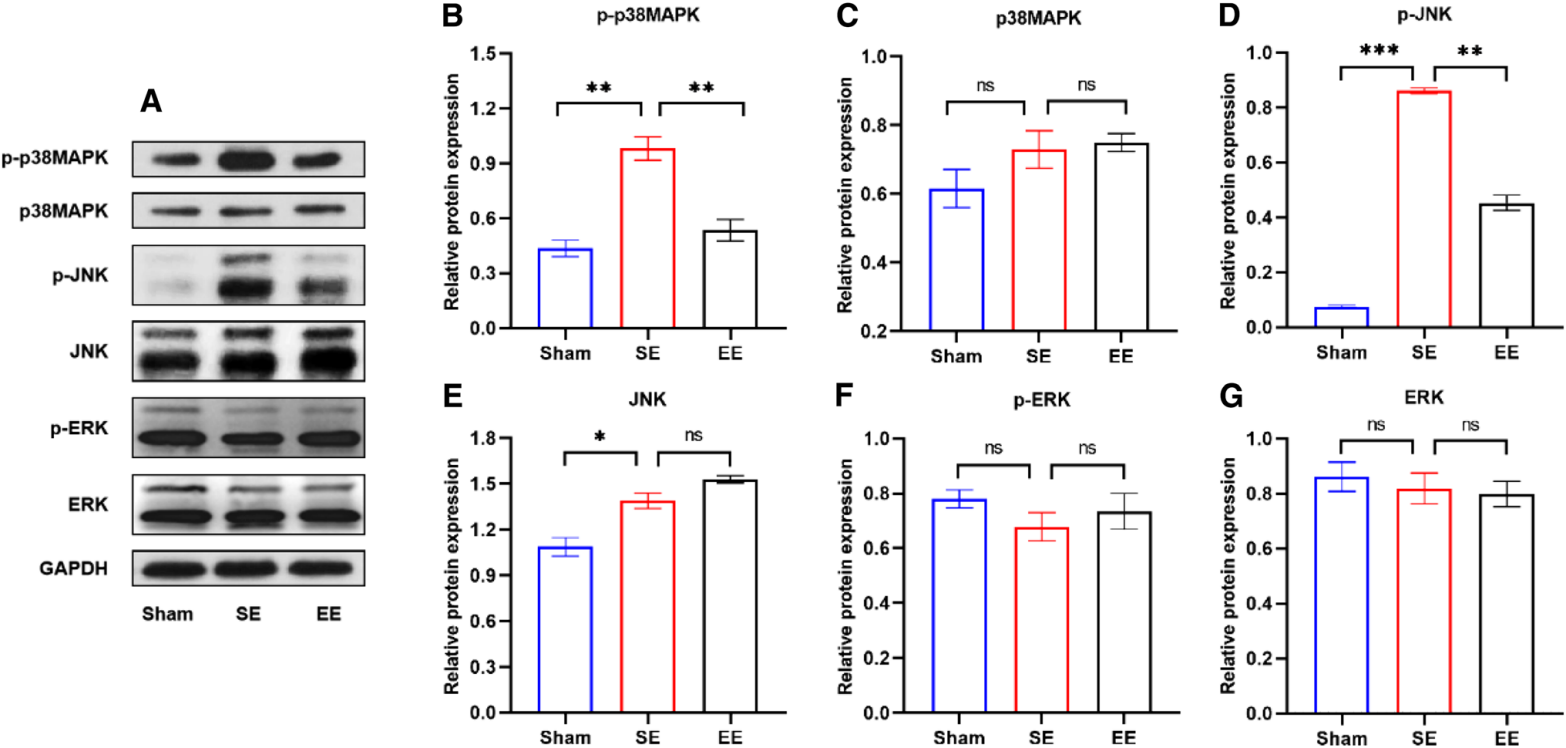
EE pretreatment inhibited the expression of MAPK inflammation related pathways. **A** The representative graph of protein band of p-p38MAPK, p38MAPK, p-JNK, JNK, p-ERK, and ERK. GAPDH was used as an internal control. **B**–**G** Quantitative results of p-p38MAPK, p38MAPK, p-JNK, JNK, p-ERK, and ERK. n = 6. Data are expressed as mean ± SEM. One-way analysis of ANOVA with Bonferroni’s post hoc test was used. *p < 0.05, **p < 0.01, ***p < 0.001

To address the above questions, we performed a comprehensive examination of the MAPK pathway. First, we detected biomarkers for the three family members. The western blot analysis results demonstrated the protein expression of p38MAPK, JNK and ERK (Fig. 4 A). Compared to the Sham group, the p-p38MAPK and p-JNK in SE group increased in varying degrees (Fig. 4B–E). Moreover, in the EE pretreatment group, p-p38MAPK and p-JNK showed a statistically significant downward trend. However, p-ERK still maintained the similar expression level as SE group (Fig. 4 F and G). This suggests that ischemia-reperfusion leads to abnormal activation of p38 and JNK, and EE pretreatment partially reversed this phenomenon. In addition, since this paper mainly studies the neuroinflammation after ischemic stroke, and JNK pathway is used for the research of cancer and apoptosis [36, 37], we will not verify JNK. Many previous studies have shown that inflammation and apoptosis can affect each other, and the synergistic increase of p38 and JNK also confirms this. Nevertheless, it is undeniable that the MAPK family is closely involved in the injury process of ischemic stroke.

### STAT1 mediates the effects of EE pretreatment on acute ischemic stroke

Microglia are the classical inflammatory response cells of the central nervous system, and its activation implies the emergence of inflammation [38]. Microglia have multiple activation pathways, and signal transducer and activator of transcription 1 (STAT1) is one of its canonical pathways, and previous studies have shown that STAT1 is closely associated with inflammatory responses [39]. Furthermore, Takagi Y et al. reported the impact of STAT1 activation in ischemic reperfusion and demonstrated that STAT1 knockout mice had better performance in the face of ischemic injury [40]. what is more important, many studies have shown that p38 affects STAT1 phosphorylation in many different ways [41]. Based on the significant change of p38 protein expression, we reasonably speculate that EE pretreatment improves neuroinflammation after ischemia-reperfusion through p38MAPK/STAT1 pathway, thus alleviating brain injury.

**Fig. 5:**
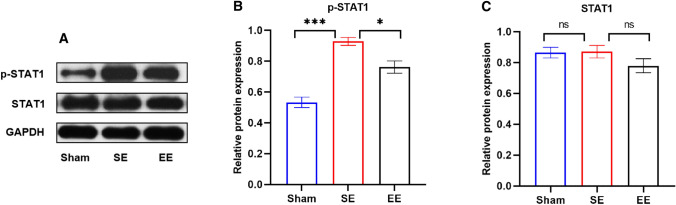
EE pretreatment inhibited STAT1 protein expression. **A** The representative graph of protein band of p-STAT1 and STAT1. GAPDH was used as an internal control. **B**, **C** Quantitative results of p-STAT1 and STAT1. n = 6. Data are expressed as mean ± SEM. One-way analysis of ANOVA with Bonferroni’s post hoc test was used. *p < 0.05, ***p < 0.001

To further confirm this hypothesis, we performed Western blot analysis on STAT1. As we expected, after ischemia-reperfusion, the protein level of p-STAT1 showed an obvious upward trend in the SE group, while the situation in EE pretreatment group was better than that in SE group (Fig. 5A–C). The above results indicated that the p38 MAPK/STAT1 pathway may be closely involved in the protective effects of EE pretreatment.

### EE pretreatment alleviated neuroinflammation in rats with acute ischemia-reperfusion

**Fig. 6:**
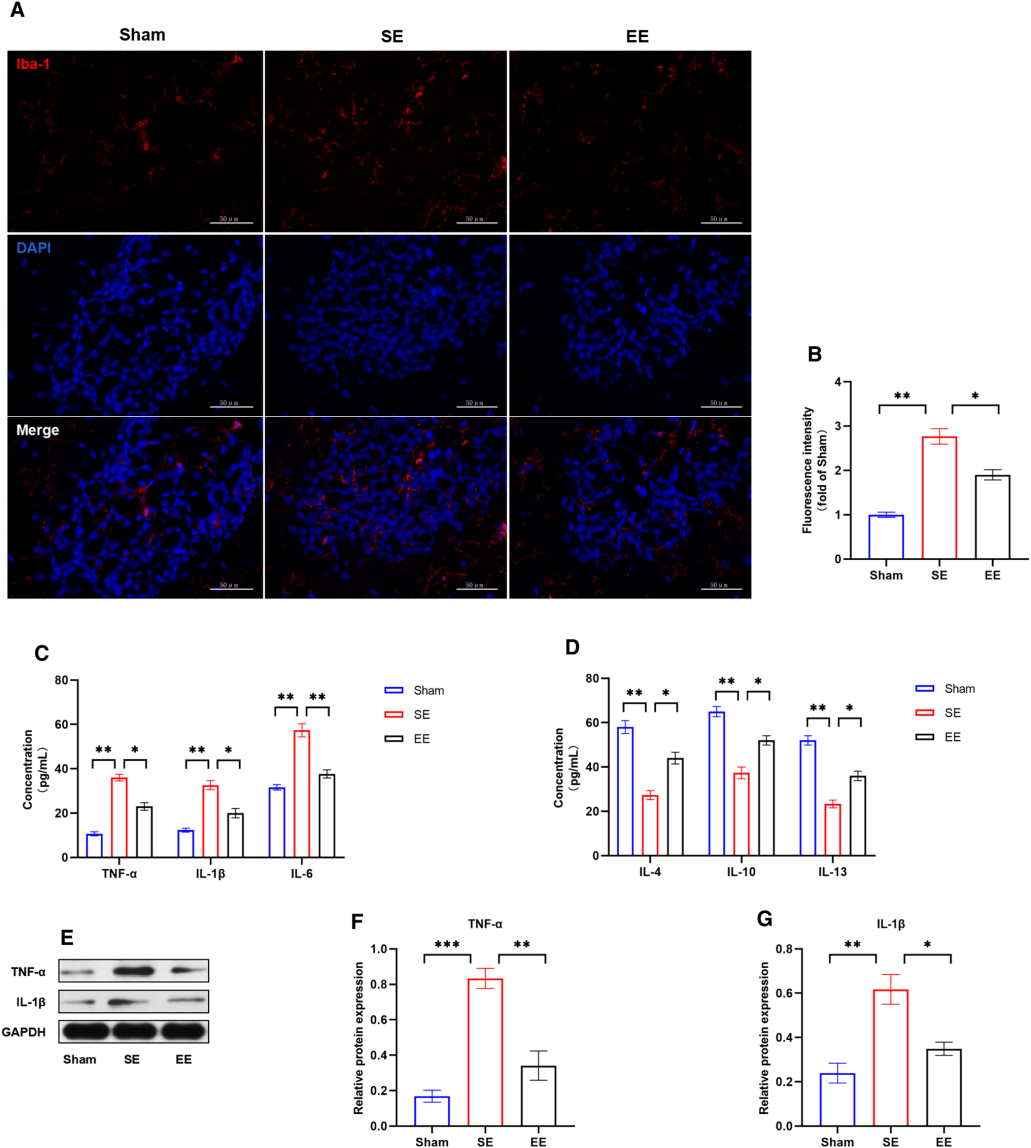
EE pretreatment alleviated neuroinflammation after ischemia-reperfusion. **A** The representative immunofluorescence images of Iba1 in ischemic penumbra of rats in each group. Scale bar = 50 μm. **B** Quantitative results of Iba1 fluorescence intensity. n = 6. Data are expressed as mean ± SEM. One-way analysis of ANOVA with Bonferroni’s post hoc test was used. *p < 0.05, **p < 0.01. **C**, **D** ELISA results of proinflammatory and anti-inflammatory factors. n = 6. Data are expressed as mean ± SEM. One-way analysis of ANOVA with Bonferroni’s post hoc test was used. *p < 0.05, **p < 0.01. **E** The representative graph of protein band of TNF-α and IL-1β. GAPDH was used as an internal control. **F**, **G** Quantitative results of TNF-α and IL-1β. n = 6. Data are expressed as mean ± SEM. One-way analysis of ANOVA with Bonferroni’s post hoc test was used. *p < 0.05, **p < 0.01, ***p < 0.001

The p38MAPK/STAT1 is an important pathway to regulate inflammation, our previous work has proved that EE pretreatment can inhibit this pathway. So we next performed the detection of inflammation related indicators. Activation of microglia is a classical hallmark of neuroinflammation onset. We first detected the activation of microglia in each group using immunofluorescence. The results showed that compared to sham operation group, SE group had the most significant microglial activation, while EE pretreatment group had significant inhibition (Fig. 6A, B). This indicates that there is less microglial activation in the EE pretreatment group after ischemia-reperfusion, which is consistent with the lighter brain injury in the EE group before.

Furthermore, the activation of p38MAPK/STAT1 pathway can promote the expression of many proinflammatory cytokines. To explore whether EE pretreatment reduces the expression of proinflammatory cytokines by inhibiting p38MAPK/STAT1 pathway, we examined the expression of classical proinflammatory cytokines in the ischemic penumbra of ischemic cortex using an ELISA Kit (Fig. 6C). After ischemia-reperfusion, the three pro-inflammatory factors were increased in different degrees in the EE group, but the increase was significantly prevented by EE pretreatment. Similarly, we also tested the related anti inflammatory cytokines. Consistent with the expected results, anti inflammatory cytokines in SE group showed a downward trend, while the EE group prevented this decrease (Fig. 6D). This indicates that EE pretreatment alleviates neuroinflammation from different aspects. To further confirm this conclusion, we performed Western blot analysis on classical proinflammatory cytokines TNF-α and IL-1β. As illustrated in Fig. 6E–G, SE group has higher expression level of proinflammatory cytokines, which was suppressed by EE pretreatment. In conclusion, the above data indicate that EE pretreatment alleviates neuroinflammation after acute ischemia-reperfusion, which may be related to the inhibition of p38MAPK/STAT1 pathway.

## Discussion

Many studies have shown that exercise may enhance motor stimulation and thereby promote recovery after stroke [42, 43]. In the 1940s, Hebb first proposed the concept of enriched environment. Compared with the standard environment (SE), the EE has small toys with different functions and shapes, and a larger activity space for exploration. Secondly, more participating members in the space provide social stimulation, and items will be changed regularly to maintain the novelty of the environment. Therefore, EE treatment is considered as one of the methods of stroke rehabilitation. The patients who stayed in the rehabilitation ward, in addition to their daily rehabilitation training, spent most of their time alone. Related clinical studies have shown that increasing the communication between stroke patients and the outside world (daily activities, communication, group activities, etc.), can maintain patients in a pleasant mental state, thereby promoting functional recovery after stroke [44–46]. However, most of the current studies on EE still focus on its therapeutic effect and related mechanisms. Few studies have clarified whether the EE in advance can affect the pathological process of stroke to elicit neuroprotection. Inflammation is closely related to the occurrence and treatment of ischemic stroke, but its underlying mechanism is still unclear. Given that both EE treatment and inflammatory responses are involved in the development of stroke, we speculated that they might be closely related to the treatment of stroke. Therefore, in this study, we studied whether the inflammatory mechanism is involved in the neuroprotection after acute ischemic stroke induced by EE pretreatment.

Our study demonstrated that EE pretreatment significantly reduced neurological deficits, brain water content and neuronal apoptosis after acute ischemia reperfusion, which indicated that EE pretreatment indeed had a protective effect. Then, we verified the classic inflammatory pathway MAPK. The results indicated that the protein expression levels of p38 MAPK were significantly different. In addition, we confirmed that MCAO promoted the activation of p38MAPK/STAT1, which was inhibited in EE pretreatment group. In order to study whether EE pretreatment induced neuroprotection is related to inflammatory response, we examined inflammation related indicators. The results of immunofluorescence showed that EE pretreatment significantly reduced the expression of Iba1, and the results of ELISA were consistent with that. These findings indicated that EE pretreatment inhibited the activation of p38MAPK and its downstream (STAT1), thereby reducing the release of proinflammatory cytokines. In brief, our research indicates that EE pretreatment increased the resistance of rat brain to disease invasion to a certain extent. When faced with acute ischemia reperfusion injury, rats in EE group showed less inflammatory reaction, less neuronal apoptosis and cerebral edema, and better neurological score.

**Fig. 7:**
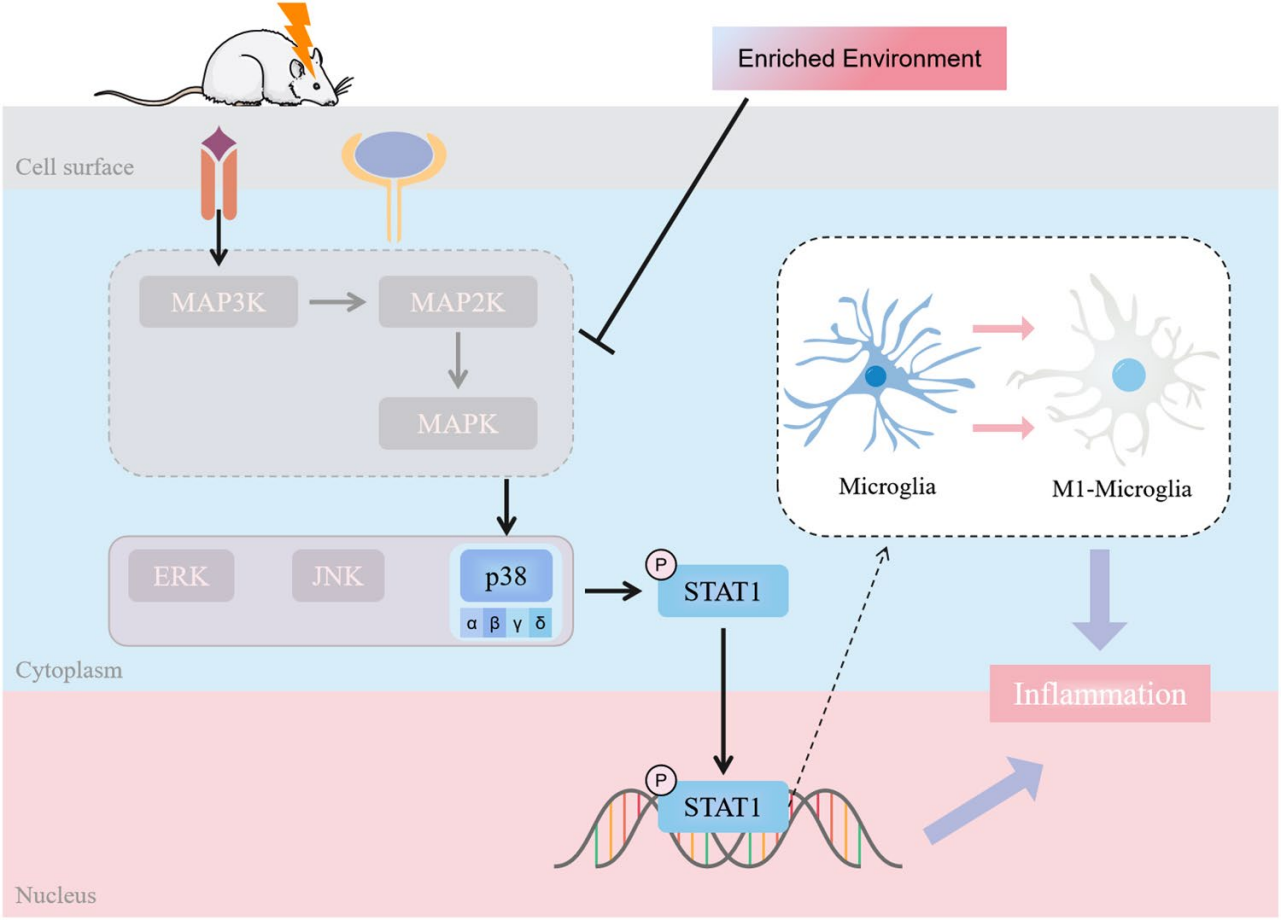
Schematic diagram of the pathway mechanism studied in this paper and the areas to be explored. Pre exposure to enriched environment alleviates brain injury after ischemia-reperfusion by inhibiting p38MAPK/STAT1 pathway

The therapeutic effects of EE have been heavily studied previously, but relevant studies on its preventive effects remain scarce. This study particularly explored the protective effects of EE pretreatment on acute ischemic stroke. Our findings contribute new ideas to the prevention and treatment of stroke. In addition, our research results also have certain significance for clinical work. First, people who live in a comfortable environment and have a good mood will have better physical fitness. Secondly, when the disease occurs, comprehensive environmental treatment similar to EE can also promote the recovery of patients. This enriched environment should include comfortable social interaction, moderate exercise, and slightly challenging work. Regrettably, this study only syudied the role of EE pretreatment on inflammatory responses, and it is worth noting that the protective effects conferred by EE pretreatment may be more than related to inflammatory responses, and responses such as oxidative stress, autophagy, and endoplasmic reticulum stress are most likely also involved, which require future studies for further exploration. Furthermore, although we have demonstrated that the p38 MAPK/STAT1 pathway is inextricably linked to the protection mediated by EE pretreatment, there is still a lot of meaningful work worth exploring and completing. First, p38 has four subtypes: p38 α, p38 β, p38 γ and p38 δ [47] (Fig. 7). At present, we cannot determine which subtype significantly affects the experimental results, and future research can focus on this. Second, there have been relevant studies that have shown that the phosphorylation of STAT1 is related to the abnormal activation of microglia. Microglia are the first major cell population to respond to injury and induce inflammation. It is reported that STAT1 is related to the activation of microglia [48, 49]. Therefore, we can reasonably speculate that the inflammation induced by p38MAPK/STAT1 pathway is closely related to the polarization of microglia (Fig. 7). That is, phosphorylation of STAT1 promotes the polarization of microglia toward the M1 phenotype, thereby exacerbating inflammation and brain damage. More importantly, this paper still has some limitations. It is well known that age is a key risk factor for ischemic stroke [50]. In recent years, the gradual increase of the incidence rate of stroke is closely related to the aging of the population. At present, most basic research in the field of stroke uses young mice. Their physical functions are in the best period, which can minimize the interference factors brought by aging. However, we cannot ignore the fact that most of the patients with clinical stroke are elderly. After stroke, a large number of cells proliferate in the capillary wall near the infarct site in old rats, and this premature cell proliferation often leads to the rapid formation of glial scar, which affects the nerve recovery and repair [51]. Because young mice usually recover rapidly in the subacute stage of stroke, and can not better reproduce the physical functions of the elderly. Therefore, the effective treatment obtained from the study on young rats should be further demonstrated in older rats. Especially in the research on riched environment, cognitive function will gradually decline with the increase of age. It is important to assess the impact of environmental factors on functional recovery in the elderly animal model. Importantly, some scholars have studied this, and their experiments show that isolation environment increases the number of inflammatory cells in the damaged area of old rats, rather than young rats [52]. The aged animals raised in the riched environment performed better than the aged animals raised in isolation. In addition, some studies also showed that after electric stimulation was used to treat the aged rats after stroke, Tubulin beta III and newborn DCX cells in the infarcted area increased significantly, concurrently, electric stimulation had a detrimental effect on the asymmetric sensorimotor deficit [53]. In conclusion, aging and co-morbidities have a major impact on the incidence, consequences and the efficacy of any intervention, and future studies on stroke should carefully consider age as a key factor.

In conclusion, our study demonstrated that EE pretreatment could protect the brain after stroke by inhibiting the p38 MAPK/STAT1 pathway. Thus, EE can be one of the most promising means of disease prevention. Secondly, p38MAPK/STAT1 pathway may be a latent target for the prevention of ischemic stroke. In the future research, we will continue to explore the relevant mechanism between EE pretreatment and inflammatory response and confirm it with the rescue experiments.

**Table 1:** Longa’s neurological function score

Score	Degree of damage	Physical activity
0	No neurological symptoms	Physical activity was normal
1	Mild focal injury	Inability to fully extend the front paw on the paralyzed side
2	Moderate focal injury	Turning around to the paralyzed side when walking
3	Severe focal injury	Tipping to the paralyzed side when walking
4	Severe injury	Unconscious, unable to walk

## Data Availability

The data in this study are available from the corresponding author on reasonable request.
